# Successful treatment of kasabach-merritt syndrome with vincristine and surgery: a case report and review of literature

**DOI:** 10.1186/1757-1626-1-9

**Published:** 2008-05-23

**Authors:** Kotb Abass, Hekma Saad, Mostafa Kherala, Alaa A Abd-Elsayed

**Affiliations:** 1 Department of Paediatrics, Maternity and Children's Hospital, Buraidah, Al-Qassim, Kingdom of Saudi Arabia; 2 Department of Paediatric Surgery, Maternity and Children's Hospital, Buraidah, Al-Qassim, Kingdom of Saudi Arabia; 3 Department of Public Health and Community Medicine, Faculty of Medicine, Assiut University, Assiut, Egypt

## Abstract

**Introduction:**

Haemangiomas are vascular lesions resulting from abnormal proliferation of blood vessels. They are the most common pediatric neoplasm. Kasabach-Merritt syndrome is a rare type of vascular lesion with peculiar characteristics. The diagnosis is based upon three basic findings; enlarging haemangioma, thrombocytopenia and consumption coagulopathy.

**Case presentation:**

A 5 month old boy was admitted to the Pediatrics department for the management of an abdominal wall mass. He was the first child of consanguineous parents, born in a private hospital following uncomplicated pregnancy and delivery. At birth a bluish birth mark 5 cm × 5 cm was noted below the umbilicus. Over the next five months, this birth mark increased in size and evolved into a swelling. As a result, the patient was admitted to Maternal and Child Health (MCH) unit for the management of this swelling.

The clinical findings and imagining studies followed by laboratory investigations strongly suggested the diagnosis of Kasabach-Merritt syndrome.

Vincristine was initiated after a trial of corticosteroids when the platelet count was 6000/cmm. One week after the start of vincristine the size of the lesion started to decrease. At the end of 6th week the lesion size decreased to half and the platelet count increased to 49,000/cmm. Vincristine was continued for another 2 weeks, no further improvement in lesion size or platelet count was observed. Vincristine was discontinued and the patient was shifted to the paediatric surgery department. A fresh platelet transfusion was given and the haemangioma was excised completely.

The histopathological examination of the excised mass revealed a caverno-capillary haemangioma with infiltration into skeletal muscles.

**Conclusion:**

Six weeks treatment with vincristine in a dose of 0.5 mg/kg/week followed by surgical excision may be the best management in selected cases of Kasabach-Merritt syndrome.

## Introduction

Haemangiomas are vascular lesions resulting from abnormal proliferation of blood vessels. They are the most common pediatric neoplasm. They have a special importance in clinical practice because of their distinct properties and behavior. Some haemangiomas are very small and hardly visible while others are large producing significant disfigurement [1]. The management of these lesions not only depends upon their size and site but also on several other distinct features as for Kasabach-Merritt syndrome, Klippel-Trenaunay-Weber syndrome, Sturge-Weber syndrome, Rendu-Osler-Weber syndrome and von Hippel-Lindau disease[1].

Kasabach-Merritt syndrome is a rare type of vascular lesion with peculiar characteristics. The diagnosis is based upon three basic findings; enlarging haemangioma, thrombocytopenia and consumption coagulopathy. The thrombocytopenia and consumption coagulopathy is known as Kasabach-Merritt phenomenon [1]. It is argued that exposure of subendothelial elements or abnormal endothelium within the haemangioma results in aggregation and activation of platelets with a secondary consumption of clotting factors [2]. If untreated, Kasabach-Merritt syndrome can be life threatening with high mortality. The present report describes a patient with Kasabach-Merritt syndrome. The clinical presentation of the case and outcome of selected treatment modalities are discussed in the light of previous studies done in connection with this subject.

## Case presentation

**Figure 1:**
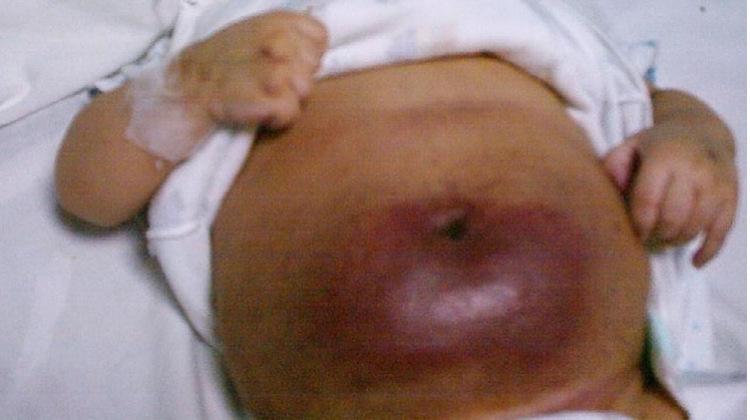
Reddish-blue lesion covering lower part of the abdomen (caverno-capillary haemangioma).

A 5 month old boy was admitted to the Paediatric department for the management of an abdominal mass. He was the first child of consanguineous parents, born in a private hospital following uncomplicated pregnancy and delivery. At birth a bluish birth mark 5 cm × 5 cm was noted below the umbilicus (figure 1). Over the next five months, this birth mark increased in size and evolved into a swelling so the patient was admitted in MCH for the management of this swelling.

Initial examination in the paediatric ward showed a playful child with normal development. His growth parameters were well within normal limits. The abdomen was distended below the umbilicus with obvious mass (12 cm × 7 cm) involving both inguinal and hypogastric areas. The mass was firm, non-tender, with indiscrete margins. The overlying skin was reddish-blue and smooth with no signs of inflammation. There was no visceromegaly. In addition skin at other areas and mucus membranes was normal. The examination of the central nervous system, cardiovascular system, and respiratory system was unremarkable.

The initial Complete Blood Count (CBC) showed a haemoglobin level of 10.6 gm %, a white cell count of 8000/cmm, and a platelet count of 94,000/cmm. The relevant haematological investigations carried out during 14 weeks of the in-patient stay and thereafter in the out-patients' department are shown in Table 1.

**Table 1:** Relevant haematological investigations carried out during hospital stay and follow-up at outpatients' department of MCH.

Hospital stay	Investigations				Treatment
	
	WBC/cmm	Hb gm %	Platelet/cmm	Others	
1^st ^day	8000	10.6	94000		
4^th ^day	11900	11.4	5000	PT > 100 sec (N = 28.8) PTT > 120 sec (N = 34.8) Fibrinogen = 19.7 mg/dl (N = 160–350)	Prednisolone
7^th ^day	9300	9.0	4000		
5^th ^week	8200	9.7	6000	PT > 100 sec (N = 28.8) PTT > 120 sec (N = 34.8) Fibrinogen = 42.3 mg/dl (N = 160–350)	Vincristine
7^th ^week	10400	10.2	19000		
9^th ^week	9500	9.8	45000		
11^th ^week	8500	10.7	49000		
13^th ^week	9500	11.2	50000		Surgery
4^th ^month (OPD)	10200	10.9	278000	PT = 16.5 sec (N = 28.8) PTT = 34.2 sec (N = 34.8) Fibrinogen = 236 mg/dl (N = 160–350)	
8^th ^month (OPD)	5900	11.0	290000	PT = 16.2 sec (N = 28.8) PTT = 27.8 sec (N = 34.8) Fibrinogen = 251 mg/dl (N = 160–350)	

OPD = Out patients' department

During the hospital stay, the liver function tests and urine examination did not reveal any abnormality. An ultrasound of the abdomen showed a fluid collection in the subcutaneous tissue and muscle planes of anterior abdominal wall. Computed tomographic scan showed an extensive heterogeneous soft tissue density mass suggesting a haemangioma.

The above clinical findings and imagining studies followed by laboratory investigations strongly suggested the diagnosis of Kasabach-Merritt syndrome.

**Figure 2:**
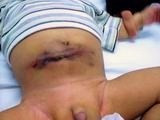
Abdominal lesion on day 7 postoperatively.

On day four of admission, the child developed mild purpuric rash. The haemotological parameters of the disease were repeated (Table 1). The platelet count was found to be 5000/cmm, haemoglobin level of 11.4 gm%, prothrombin time more than 100 seconds, partial prothrombin time more than 120 seconds and fibrinogen level of 19.7 mg/dl. Platelet transfusion and fresh frozen plasma were given and prednisolone was started at a dose of 2 mg/kg/day in 3 divided doses. This therapy was continued for four weeks and then tapered off in another four weeks period without any response. During this period the patient received 4 units of platelet transfusions, 3 units of packed red cell transfusions and 3 units of fresh frozen plasma transfusions for supportive measures. The eight weeks of steroid therapy resulted in the development of cushanoid features. As the steroid was decreased in dose, vincristine was instituted at dose of 0.5 mg/kg/week. When vincristine was initiated the platelet count was 6000/cmm. One week after the start of vincristine the size of the lesion started to decrease. At the end of 6^th ^week the lesion size decreased to half and platelet count increased to 49,000/cmm. Vincristine, in the same dose, was continued for another 2 weeks, no further improvement in lesion size or platelet count was observed. Vincristine was discontinued and the patient was shifted to the paediatric surgery department. A fresh platelet transfusion was given and the haemangioma was excised surgically completely (figure 2). Two blood transfusions were given post-operatively to correct the haemoglobin level and other blood parameters. The histopathological examination of the excised mass revealed a caverno-capillary haemangioma with infiltration into skeletal muscles of the abdominal wall. Two, four and six month follow-up after surgery shows complete recovery with total correction of haemotological parameters. (Table 1)

## Discussion

Kasabach-Merritt syndrome shows wide variation in its response to different treatment modalities. Currently, there are no known treatment guidelines [3]. Different interventions are recommended including compression, embolization, use of interferon, use of steroids, laser therapy, sclerotherapy, chemotherapy, radiation or surgery. [1,2,4]. In each case the treating physician must decide the most suitable treatment to achieve maximum involution of the lesion and preservation of organ function.

Several researchers agree that most patients with Kasabach-Merritt syndrome respond to steroids within a few days of treatment [5]. However, one third will not respond to conventional dose of prednisolone (2 mg/kg/day) and mega dose 5 mg/kg/day may be effective. The present patient did not respond to conventional dose of steroid. Due to patient's condition the mega dose was not used and the treatment was switched to vincristine.

The angiogenetic character of Kasabach-Merritt syndrome indicates that chemotherapy is a logical treatment. Enjolras and associates have reported that several steroid non-responders show dramatic response to vincristine[6]. A multicenter study in the United States also showed that vincristine is a safe and effective treatment in the management of Kasabach-Merritt syndrome [7]. However, it is recommended that vincristine should be added when other modalities are unsuccessful. In the present case the vincristine was well tolerated but the response to this treatment was partial.

Enjolras et al. found that the response rate to chemotherapy was 100% with an average duration of treatment being 22 weeks. In the present case it was not possible to continue chemotherapy as the response became static after 6 weeks. The potential side effects of vincristine such as irritability, loss of deep tendon reflexes, and abdominal pain were observed by Enjolras et al. but were not noted in this case [6].

While monitoring the effects of above treatments, prednisolone and vincristine, the outcome measures were an increase in platelet count and fibrinogen level, and decrease in tumor size. Similar parameters were also used in other studies [7]. Haisley-Royster and co-workers have reported that the increase in platelet count precedes the regression in tumor size [7]. This phenomenon was also observed in the present case.

Despite the risk of malignancy, radiation therapy is still used for the treatment of Kasabach-Merritt syndrome [8]. Other researchers recommend the use of interferon alpha when steroid treatment fails [9]. Some researchers have used combinations of two or more treatment modalities [5], while other investigators recommend a stepwise multimodal approach for the treatment of this disease entity [10,11].

A major mainstay of the treatment of Kasabach-Merritt syndrome is surgical excision. This approach is recommended for single cutaneous lesions or multiple lesions in the spleen (splenectomy) or liver (wedge resection/hepatectomy) [2,12,13]. This is the only treatment that provides cure in significant number of cases. However, before each surgical intervention the patient must be stabilized. In the present case the administration of vincristine helped to stabilize the patient's general condition which was not possible with sole and repeated administrations of platelets and fresh frozen plasma.

## Conclusion

Six weeks treatment with vincristine in a dose of 0.5 mg/kg/week followed by surgical excision may be the best management in selected cases of Kasabach-Merritt syndrome.

## Competing interests

The authors declare that they have no competing interests.

## Authors' contributions

KA & HS carried out the patient diagnosis, investigation, chemotherapy, follow up, management of medical emergencies during surgery and provided medical care in the ICU, MK performed the surgery and removed the mass, AAAE general coordination, drafting of the manuscript, writing the final manuscript and provided important suggestions

All authors read and approved the final manuscript.

## Consent

Written informed consent was obtained from the parents of our patient for publication of this case report and the accompanying images.
